# Implementation of a competency-based medical education approach in public health and epidemiology training of medical students

**DOI:** 10.1186/s13584-017-0194-8

**Published:** 2018-02-20

**Authors:** Rachel Dankner, Uri Gabbay, Leonard Leibovici, Maya Sadeh, Siegal Sadetzki

**Affiliations:** 1 Unit for Cardiovascular Epidemiology, The Gertner Institute, Chaim Sheba Medical Center, Ramat Gan, Israel; 2 Department of Epidemiology and Preventive Medicine, School of Public Health, Sackler Faculty of Medicine, Tel Aviv University, Tel Aviv, Israel; 3 Quality Unit, Rabin Medical Center, Petah Tikva, Israel; 4 Department of Medicine E, Beilinson Hospital, Rabin Medical Center, Petah-Tiqva, Israel; 5 Sackler Faculty of Medicine, Tel Aviv University, Tel Aviv, Israel; 6 Cancer & Radiation Epidemiology Unit, Gertner Institute, Chaim Sheba Medical Center, Ramat Gan, Israel

## Abstract

**Background:**

There is increasing agreement among medical educators regarding the importance of improving the integration between public health and clinical education, understanding and implementation of epidemiological methods, and the ability to critically appraise medical literature. The Sackler School of Medicine at Tel-Aviv University revised its public health and preventive medicine curriculum, during 2013–2014, according to the competency-based medical education (CBME) approach in training medical students. We describe the revised curriculum, which aimed to strengthen competencies in quantitative research methods, epidemiology, public health and preventive medicine, and health service organization and delivery.

**Methods:**

We report the process undertaken to establish a relevant 6-year longitudinal curriculum and describe its contents, implementation, and continuous assessment and evaluation.

**Results:**

Central competencies included: epidemiology and statistics for appraisal of the literature and implementation of research; the application of health promotion principles and health education strategies in disease prevention; the use of an evidence-based approach in clinical and public health decision making; the examination and analysis of disease trends at the population level; and knowledge of the structure of health systems and the role of the physician in these systems. Two new courses, in health promotion, and in public health, were added to the curriculum, and the courses in statistics and epidemiology were joined. Annual evaluation of each course results in continuous revisions of the syllabi as needed, while we continue to monitor the whole curriculum.

**Conclusions:**

The described revision in a 6 year-medical school training curriculum addresses the currently identified needs in public health. Ongoing feedback from students, and re-evaluation of syllabus by courses teams are held annually. Analysis of student’s written feedbacks and courses evaluations of “before and after” the implementation of this intervention is taking place to examine the effect of the new curriculum on the perceived clinical and research capacities of our 6-year students.

## Background

Among the profound changes that have occurred in the practice of medicine in the twenty-first century are greater sophistication, high-technological dependence, a personalized approach and extreme increases in costs. Modern preventive medicine uses proactive interventions, surgery and chronic use of preventive medications. Clinical reasoning and clinical decision-making have expanded from being almost exclusively based on deterministic pathophysiological principles to include clinical and population-based evidence [1]. Current medical practice is also multi-disciplinary, mandating coordinated teamwork. The need for stronger links between medicine and public health is ongoing, and includes the need for a clinical and public health workforce trained to collaborate in a multi-disciplinary environment [2, 3]. Increasingly complex epidemiological research methods require physicians to acquire broad competencies in research methodologies and statistics to enable their critical appraisal of the literature when making clinical decisions. Physicians’ use of evidence-based medicine (EBM) has gained importance for weighing benefits and harms of clinical decisions such as relating to diagnoses, disease prognosis and intervention.

In parallel to the above, many changes have occurred in medical education. Medical training has shifted from frontal teaching and an observer-apprentice approach to a task oriented approach [4]. Recommendations of the 2010 Carnegie report, which are being implemented in the US and the UK, include for example, the need to strengthen connections between formal and experiential knowledge across the continuum of medical education [4]. In addition, up-to-date teaching should emphasize an evidence-based approach that empowers the medical student to actively search, rank, appraise, interpret and implement the evidence that is relevant to individual patients [5].

Preventive medicine, which is often the most cost-effective medical approach, has become mandatory, to restrain the increasing costs of chronic disease care. For many years, public health was a marginalized low profile discipline in medical education [6]. However, there is growing concern among medical schools of gaps in knowledge and competence of physicians in areas such as clinical preventive services, quantitative methods of risk and outcomes assessment, the practice of community medicine, and health services organization and delivery [7, 8]. Consequently, several organizations including the Association of American Medical Colleges, the Institute of Medicine (IOM), and the United Kingdom General Medical Council (GMC) have emphasized the importance of undergraduate medical training in the field of public health [3, 9–11]. The effect of physician’s health care practice on patients’ health care practice was demonstrated in the positive relationship found between physicians and patients in influenza vaccination rates [12].

The Sackler School of Medicine at Tel-Aviv University was founded in 1964 with the goal of educating highly professional, knowledgeable and compassionate physicians. In accordance with the above-mentioned concerns, and as part of the implementation of a revised curriculum, a committee of medical doctor faculty members who are board certified in public health and experienced in epidemiological research, was convened in 2012–2013. The task of the committee was to evaluate and update objectives for the public health curriculum for medical students; to review and revise the current curriculum; to introduce a revised curriculum in public health; and to introduce appropriate teaching methods in accordance with the competency-based medical education (CBME) approach [13]. This paper presents the process and recommendations of the committee, which were approved and adopted by the teaching committee of the Tel Aviv Sackler Medical School, and implemented during the past 4 years.

### Training medical students in public health

Awareness has grown over the past 2 decades, to the importance of the public health discipline to clinicians, and to the need to instill medical students with competencies in public health [14]. The Consensus Conference on Undergraduate Public Health Education advocated that all undergraduate medical students have access to an education in public health [3]. The Association of American Medical Colleges and The Healthy People Curriculum Task Force published recommendations to include a population health curriculum as part of the 4 years of medical training [3]. The IOM has since called for the US public health system to evolve from a government-centered system to involve broad partnerships with healthcare and other organizations in communities [15, 16]. In the working document, ‘Tomorrow’s Doctors’ [17–19], the UK GMC recommended that medical school education include education in disease prevention, sociological and psychological aspects of health and disease, population health, scientific research methods and critical appraisal of the literature [18]. Medical schools in the US and the UK have been placing greater emphasis on the teaching of clinical prevention and health promotion [3, 19]. The need to dedicate a specific curriculum for the aspects of how the health system functions and what the role of the clinician in this system was recently recognized by the AMA educational consortium, which published a book on health systems science in medical education, calling to bring forth the “third pillar”, which was until now “part of the hidden curriculum in medical education”, intertwining with the other two (traditional) pillars: basic science and clinical science [20]. The understanding of how physicians deliver care to patients, how patients receive care, and how health systems function, are recognized as a pillar which necessitate medical students training as part of the need to align medical education with the ongoing changes in health care delivery.

### Examples of changes over the last decades in the curricula of public health training in several medical schools around the world

#### Competencies in statistics and epidemiology as tools for conducting and understanding quantitative medical research

A historical view of statistics training was that physicians need to know statistics primarily if they were conducting or going to conduct research during their medical career; and when conducting research, they could generally rely on professional consultation with statisticians [21]. Nowadays, physicians use statistics and probability methods for a wide range of activities [22]. Statistics and related competencies are used in daily clinical practice for understanding the validity and precision of study results, explaining risk to patients, comparing treatment protocols and outcomes, interpreting the relevance and implications of diagnostic test results, interacting with drug representatives and reading pharmaceutical literature [10]. Physicians need to be capable of interpreting clinical epidemiology data and of understanding the limitations of research and statistical inference. The sophisticated statistical methods that are used in an increasing number of studies necessitate good understanding of statistics to appraise the scientific literature. Surveys conducted in various countries show a need for improving skills of epidemiological research, statistical inference and data analysis among physicians and medical students [23–26]. Almost half of UK physicians who responded to a questionnaire felt that statistics training did not seem useful during their attendance at medical school; however, 73% felt that statistics were relevant to their subsequent careers and that teaching statistics should include lectures, seminars and problem-based practical exercises [10]. The authors recommended that statistical training should start early and continue throughout medical school; and be presented at an understandable level, which is practical and integrated with other subject areas [10].

During the 1960’s at Harvard Medical School there was a long-running required Biostatistics course. By the 1970s there was an elective course, taken by a third of the class that was called, “Introduction to Biostatistics and Epidemiology.” By the early 1980s a clinical-decision making course was added; and today that same course would be called “Evidence Based Medicine” (EBM). In the last decade, Harvard Medical School implemented a course for first-year medical and dental students entitled “Clinical Epidemiology and Population Health” [9]. The objectives of the course were to instill knowledge in basic epidemiology and biostatistics, causal inference, confounding and other issues related to research interpretation, decision making and skills for clinical and population-level interventions, health promotion and behavior change strategies, physicians’ roles in the public health system and population level surveillance.

A few years ago, the University of Toronto initiated a 4 year course for undergraduate medical students, which broke down the barrier between the pre-clerkship period and clinical clerkships [27]. Based on a longitudinal, “spiral” curriculum, the course revisits educational concepts at increasing levels of complexity across the curriculum. Descriptive epidemiology is taught in the first year, analytic epidemiology in the second year and clinical epidemiology in the third and fourth years. Similarly, the basic structure of the healthcare system is taught in the first year; then a project involving organization of community-based services in the second year; quality improvement and patient safety in the third year; and the effect of physicians’ payment systems on quality of patient care in the fourth year. After the change in the organization of the course material into the longitudinal curriculum with no change in the number of hours of learning, the ranking of the University of Toronto’s training in public health improved and became number one among all medical schools in Canada.

#### Evidence based medicine

The early introduction of EBM in medical schools has been effective in changing the thought process of the medical graduates. It was also found to increase the ability for logical and critical appraisal, better suited for the understanding of the disease process and subsequent management [28]. In England, a six-week full time course linking EBM with ethics and the management of change in health services was introduced for third-year undergraduate medical students in Imperial College London [29]. The students undertook projects such as hand washing in a neonatal unit to prevent infections, drug monitoring in the elderly to reduce the risk of falls, and the use of peak flow meters in the management of asthma. The course supported the notion that undergraduates and junior clinical students can adopt and promote significant changes that make clinical care more evidence-based.

#### Health promotion

Health Promotion is a resource for theoretical knowledge and practical skills in health issues, such as sexual health, nutrition, physical activity, exercise and fitness, weight control, and alcohol and tobacco control. In 2010, less than half of the schools in the UK included sports and exercise medicine as part of their curriculum. King’s College London introduced exercise medicine, which focused on the health benefits of physical activity, the doctor’s role in assessing and prescribing physical activity, and the physiological adaptations and risks of physical activity [30]. The intervention significantly improved the confidence of preclinical medical students in their ability to counsel patients on the health benefits of physical activity, as well as their knowledge of recommended physical activity guidelines [30]. Medical students who underwent obesity intervention education scored higher on relevant knowledge, had more self-confidence in physical activity and nutrition counseling, and took more waist-hip measurements [31]. In a community health center serving a Latino immigrant population in the United States, a 9-month pilot course for medical students that combined didactic instruction in the social determinants of health with practical experience in developing, implementing and evaluating an intervention was shown to be feasible and effective [32].

Summarizing the above, the urgent need to strengthen the education of medical students in the field of epidemiology and public health in an integrative manner during the pre-clinical and clinical years, has become evident in many countries and action has been taken. Several challenges have had to be met, including the “old” perception that this topic is of little relevance to clinical practice, low funding, low institutional priority and the competition with other traditional fields (e.g. anatomy, physiology, biochemistry and histology) [33]. Nonetheless, recognition of the importance of this field has increased dramatically [34].

### Findings and insights

#### The experience of Sackler Faculty of Medicine in the adoption implementation and evaluation of competency-based medical education in public health

A committee was appointed in 2012 to propose a competencies oriented curriculum in public health for medical students. Our form of action was multistep, much like the Situational Model [35] starting with mapping the courses provided by our department (the department for Epidemiology and Preventive Medicine) to the curriculum of the 6-year medical training. In parallel, we defined the required competencies, expected from a medical student and a clinician, in public health. We then looked into each course syllabus and pointed at gaps as well as overlaps between courses. Finally, we proposed a revised curriculum in public health that incorporates all of our conclusions and suggestions. This was presented to the Faculty of Medicine’s Educational Committee and approved by the Dean after adjustments were made according to the Faculty’s constraints. We continuously review the courses’ evaluations students voluntarily and anonymously fill in the Web-based university portal, and modify the courses accordingly.

##### Defining the required competencies

The committee defined 3 main goals of training of medical students according to their future needs and responsibilities: a) critical appraisal of the scientific literature to inform practice; b) conducting research using epidemiological tools and methods; and c) practicing and advocating health promotion and disease prevention in the clinic. Following these goals the main competencies physicians require were defined:

- Skills to appraise the quality of the various types of epidemiologic research and to acquire tools for comprehensive reading and understanding scientific literature according to EBM;
- Competency in efficient and precise literature search;
- Competency in basic statistical skills;
- Competency in planning and conducting research, i.e. knowledge of epidemiological methods including the various study designs, choice of an appropriate study population, methods for data collection, analysis and interpretation of study results;
- Competency in applying health promoting principles and strategies in the selection of disease prevention measures and recommendations;
- Competency in implementation of EBM techniques in public health decision making, e.g. immunizations and population screening; and
- Competency in examining and analyzing disease trends from a population perspective.
- In addition, we identified the importance of understanding the structure of health systems and of increasing the awareness of the role of the physician in these systems as a means of better pursuing the skill of practicing and advocating health promotion and disease prevention in the clinic.

##### Identifying gaps and needs to meet the required competencies

The committee performed an overview of all relevant education and training syllabus at the Sackler School of Medicine of the Tel-Aviv University. All lectures in each course were reviewed and overlapping topics given in more than one lecture were identified. This process also enabled detecting important topics that were absent in the curriculum. The committee met all teachers and instructors and reviewed the courses syllabus with them. Those with overlapping lectures were asked to meet and revise their courses so that no unnecessary overlaps persisted. Two new courses were planned to fill in the gaps in important topics. The entire 6 year curriculum was presented and approved, first to the faculty of the School of Public Health, and then to the faculty of Sackler School of Medicine (see Table 1).

**Table 1:** The curricula before and after implementation of the revisions according to the year of medical school

Year	Before		After	
	Course name	Total number of hours	Course name	Total number of hours
1	Statistics	78	Epidemiology, Statistics, and Research methods	104
2	Epidemiology	39	–	–
2	–	–	Health promotion: The physician’s role	26
3	Evidence-Based Medicine (EBM)	16	Tools for practicing evidence based medicine	16
3	–	–	Selected paradigms in epidemiology and public health	32
3	Use of epidemiologic methods in clinical decision making	10	Use of epidemiologic methods in clinical decision making	12
4	–	–	E-learning course in planning and writing research proposals for the M.D. thesis	4
5	Principles in planning and writing a research proposal	6	Personal meeting with the M.D. students to critically review their proposal for an M.D. thesis	30 min
6	Clerkship in public health and epidemiology	36	Clerkship in public health and epidemiology	36
	Total Hours	185	Total Hours	230.5

##### Implementing the competency –based medical education approach

The new public health curriculum in our medical school is based on a longitudinal approach and was designed to harmonize and integrate the clinical and public health teaching to increase relevance, and to address the above-mentioned competencies. The public health curriculum starts early in the first year of medical school and progresses systematically, with each year building on competencies already gained. The goal is efficient utilization of time and avoidance of repetitions. The limited timeframe allocated to public health training within the busy and competitive medical school curriculum is a constraint of the program.

**Fig. 1:**
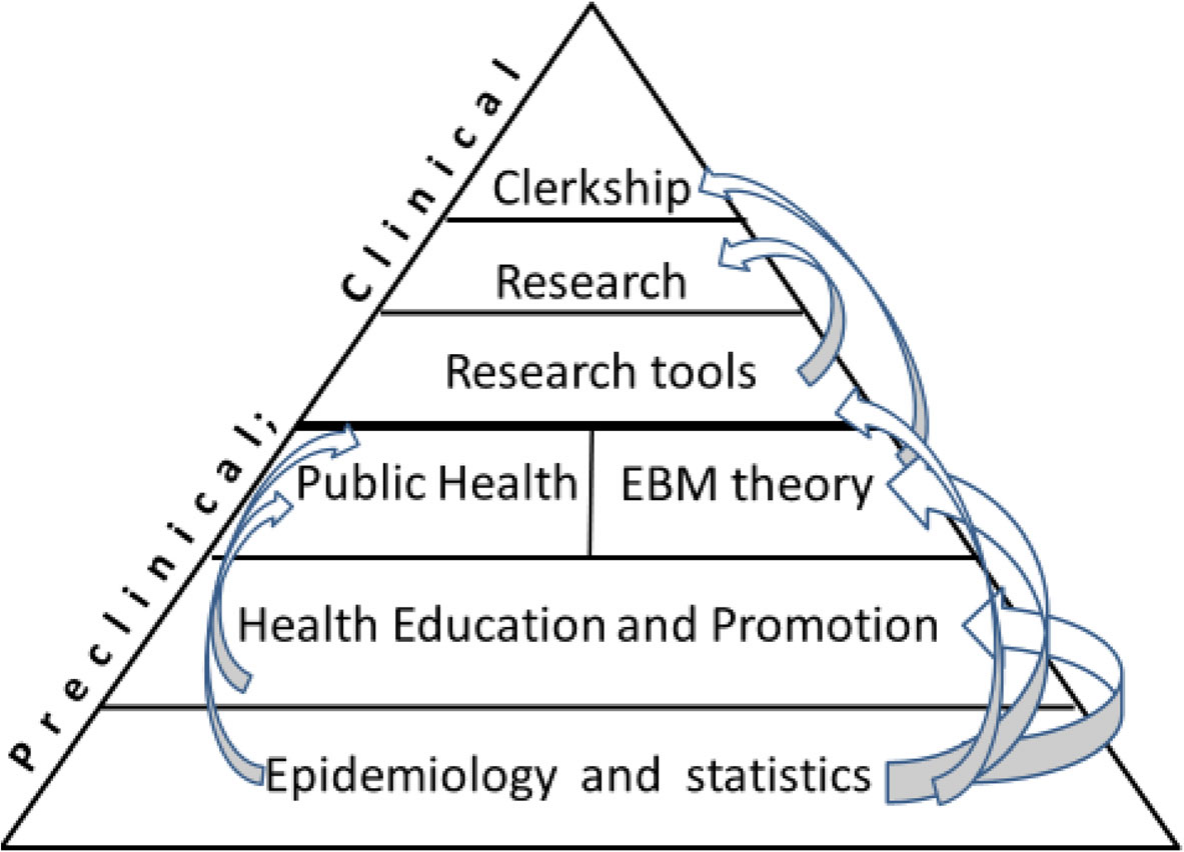
Pyramid of the Public Health curriculum

The courses and skills provided in the longitudinal public-health curriculum as part of the 6 year medical training of the Sackler Medical School are the following (see Fig. 1, illustrating the concept that epidemiology and statistics are the foundation, and are given a substantial number of hours in the curriculum, on which medical students are gradually building their public health knowledge, with the number of hours gradually decreasing yet the topics learned are more sophisticated, so that in their last year a relatively smaller, albeit very important, part of the clerkships will draw on this learning):

1. Epidemiology, statistics, and research methods (1st year): this course was re-designed to achieve a comprehensive and integrative understanding of key epidemiologic and biostatistics methods. The goals of the course are to improve students’ abilities to understand and interpret epidemiological studies and to provide practical experience in epidemiological research, study design, and key methods in biostatistics. Topics covered in the course include: the ability to integrate information and data, build statistical models, conduct data analysis, and acquire tools for decision making in selecting diagnostic tools and treatment protocols. Also emphasized are implementation of statistical and epidemiological tools for understanding disease risk and prevention, etiology and prognosis, and evaluating the success and clinical relevance of preventive interventions. The fundamentals of biostatistics and epidemiology are taught together, highlighting the relevance of these two disciplines to the understanding and interpretation of medical data.
2. Health promotion: The physician’s role (2nd year): This is one of two courses initiated following the committee’s detection of gaps in training medical students. Using epidemiological concepts and terms acquired during the first year, students are introduced to the main concepts, principles, and methods of health promotion at the individual and population levels. Students practice communicating and marketing healthy lifestyle to patients and gain knowledge of the impact of a health promoting environment (e.g. media campaigns, regulatory tools at the local and the national levels) on adoption of a healthy lifestyle. The course started as an 8-week short course but was broadened during the year 2015–2016, to include three sessions on exercise and physical activity: the approach to medical examinations before starting a physical activity program in healthy and diseased patients; the responsibility of the physician to evaluate the level of physical activity of their patients and to encourage them to exercise (Hoffman, et al. 2016); and the comprehensive physical activity prescription, which is a required responsibility of physicians to be provided to each of their patients who enters an exercise program (Joy, et al. 2016). This last session includes the students’ writing their own exercise prescription and a practical experience in training according to this prescription. An additional topic is a two lecture session in oral hygiene and its association to systemic diseases and medications.
3. Selected paradigms in epidemiology and public health (3rd year): Following the basic course in epidemiology and biostatistics in the first year, this intensive one-week course gives an overview of the epidemiology of specific diseases and conditions such as cancer, cardiovascular disease, diabetes, infectious diseases, geriatric and childhood diseases, maternal and child health, and psychiatric illnesses. The course emphasizes the specific methodologies used for the study of these illnesses and conditions and presents the specific disease registries available. The second part of the course focuses on the national health system, and aims to elucidate the role of the clinician as a public health promoter in the national health system. The paradigm of combining health policy with clinical decision making is emphasized, using relevant and timely examples.
4. Tools for practicing Evidence Based Medicine (EBM) (3rd year): Tools and techniques are provided for practicing EBM, by means of workshops and simulations of real life situations. At the end of the course, the student should be able to frame a clinical question in view of a specific clinical situation, search the medical literature, obtain the most relevant material, and critically appraise the literature so as to achieve the best available solution to the clinical question. This course reinforces the competencies provided in the first and second years and requires the student to apply them.
5. The use of epidemiologic methods in clinical decision making (3rd year): This course provides the epidemiological background to the major body organs and systems taught in the third and fourth years, while focusing on how epidemiology is used for clinical decision-making. Specific examples are presented from body systems such as the gastrointestinal and urinary tracts. The course is intended to reinforce skills covered in the first year, while exploiting the advanced stage attained in the students’ basic medical knowledge.
6. E-learning course in planning and writing research proposals for the M.D. thesis (4th - 6th year): This electronic course is designed to provide students with the necessary competencies to develop research questions and to formulate the research methodology relevant to their MD thesis. The course is built on the knowledge and capabilities of implementing the competencies taught during previous years; and it is presented through a set of online guided tools.
7. Clerkship in public health and epidemiology (6th year): Experiential learning in EBM in public health. During this 1 week interactive workshop the students experience the implementation of epidemiological tools from data collection and analysis to public health planning and decision making. The course includes practical examples such as prevention of cervical cancer or the implementation of various programs for secondary prevention of breast cancer and their impact on breast cancer mortality. As in other clinical clerkships, the students experience the process of decision making. In this case it relates to decisions in public health. At this stage, just before graduation, the students have most of the medical knowledge they will acquire during their MD degree. They have the ability to use clinical and epidemiological competencies to understand the broad range of considerations involved in health policy at the individual and population levels.

##### Program evaluation

The revised public health curriculum was implemented with first year students during 2013–2016/17. We have been revising and refining the courses of the first, second and third years according to feedback from students and lecturers. All courses in our school have a computerized feedback system, which is opened from the last lecture till the final exam, and is filled on a voluntary anonymous basis. In addition, meetings are held with the students’ representatives to discuss their expectations and feedback, and an attempt to integrate necessary changes in the courses is continuously performed. In the coming academic year (2017–18) the last class from the old curriculum will graduate. At the end of this year we will conduct a survey among these students during the clerkship in public health to evaluate their perceived understanding of public health topics and of the competencies we intended to convey in our curriculum. We will repeat this survey among the following class – the first to experience the full 6-years revised curriculum, and compare the results. In the future we intend to assess the quality of MD theses submitted at graduation, according to exposure to the intervention, and to compare evaluations of EBM skills during clinical clerkships. We expect more MD theses to be published as papers in peer-reviewed international journals.

## Conclusions

Public Health topics have been taught by the Division of Epidemiology and Preventive Medicine ever since the Sackler School of Medicine was established. The curriculum evolved over the years “bottoms up” and when a decision was made to implement a Competency Oriented approach to the medical curriculum at large we revised our curriculum. The Sackler School of Medicine committee re-designed a comprehensive curriculum in epidemiology and public health, which covers the range of topics central for current medical students’ education in those fields. Among its goals, the revised curriculum focuses on competencies required to critically appraise medical scientific literature. The curriculum has been implemented and fits the national system of medical education, which spans over 6 years of training. Our longitudinal curriculum is based on the need for a competency-based medical education (CBME) approach and an emphasis on research methods in statistics and epidemiology, preventive medicine and the application of population health principles in medical education. This is in line with the international move towards improved integration in medical training, of public health concepts, practice and research methods.

Our intended outcome is that medical school graduates will be curious and have the motivation and competencies to obtain the evidence based information they need to provide scientifically sound care to their patients; that they will have the skills to conduct research and for critically evaluating existing evidence; and that they will maximize their role in disease prevention and healthy lifestyle promotion. By having developed a longitudinal exposure for students, they are reminded at all stages of their medical education about the importance and relevance of the sciences as the basis of medical knowledge and evidence as the basis for better medical care, prevention, and public health.
